# Efficient Degradation of Recalcitrant Pharmaceuticals in Greywater Using Treatment of MBR and Immobilized TiO_2_ Porous Layers

**DOI:** 10.1021/acsestwater.4c00618

**Published:** 2024-11-29

**Authors:** Bukola Ojobe, Idris Okeowo, Jiri Rathousky, Libor Brabec, Tereza Marikova, Eliska Mikyskova, Jana Kofronova, Radek Vurm, Stepanka Smrckova, Saeed Jamali Ashtiani, Karel Friess, Zbynek Dzuman, Vojtech Kouba, Jan Bartacek

**Affiliations:** † Department of Water Technology and Environmental Engineering, University of Chemistry and Technology Prague, Technicka 5, 166 28 Prague, Czech Republic; ‡ Center for Innovations in the Field of Nanomaterials and Nanotechnologies, J. Heyrovsky Institute of Physical Chemistry, Czech Academy of Sciences, Dolejskova 3, 182 23 Prague, Czech Republic; § Forensic Laboratory of Biologically Active Substances, Department of Chemistry of Natural Compounds, University of Chemistry and Technology Prague, Technicka 5, 166 28 Prague, Czech Republic; ∥ Department of Environmental Chemistry, University of Chemistry and Technology Prague, Technická 5, 166 28 Prague, Czech Republic; ⊥ Department of Physical Chemistry, University of Chemistry and Technology Prague, Technická 5, 166 28 Prague, Czech Republic; # Department of Food Analysis and Nutrition, University of Chemistry and Technology Prague, Technická 5, 166 28 Prague, Czech Republic

**Keywords:** greywater, titanium dioxide, photocatalysis, pharmaceuticals, micropollutants

## Abstract

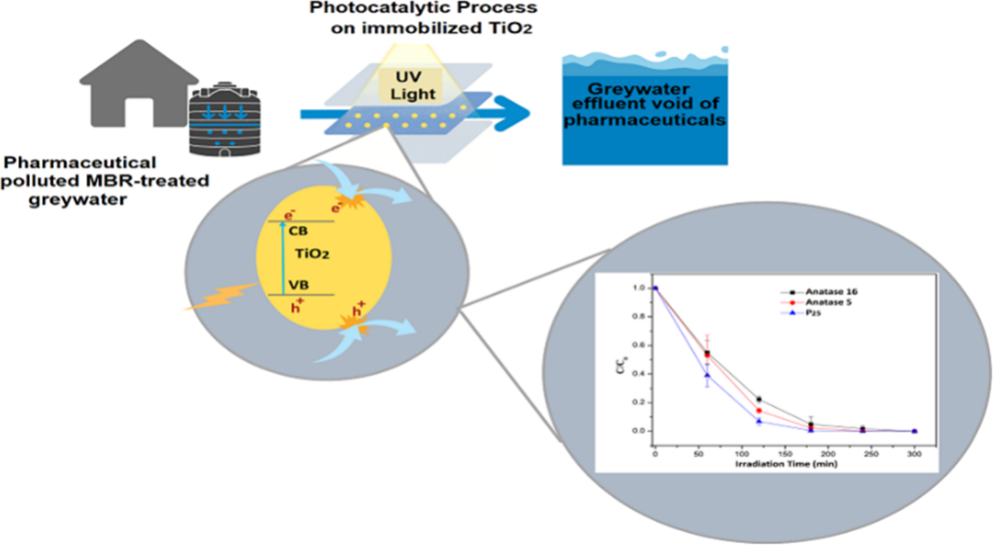

Traditional wastewater treatment often fails to remove pharmaceuticals, necessitating advanced solutions, such as TiO_2_ photocatalysis, for post-treatment. However, conventionally applied powder TiO_2_ can be cumbersome to separate from treated water. To solve this issue, this study immobilized three TiO_2_ photocatalysts (Anatase 16, Anatase 5, and P25) into porous layers and evaluated their efficacy for the degradation of three pharmaceuticals (naproxen, NPX; sulfamethoxazole, SMX; metformin, MTF) in standard solutions and greywater pretreated in a membrane bioreactor (MBR). In standard solutions, photocatalysis tests revealed a high degradation efficacy (NPX 100%, SMX 76–95%, MTF 57–75%) and challenged the belief that OH^•^ is the predominant reactive oxygen species (ROS). The primary ROS were ^1^O_2_ for NPX and OH^•^ for SMX and MTF. The raw greywater (NPX, SMX, MTF – 0.5 mg·L^–1^) treatment in MBR removed only 17–22% of the pharmaceuticals, highlighting the need for post-treatment. Using this pretreated greywater, P25 layers excelled for NPX (78 ± 5%) and SMX (73 ± 4%) but were less effective for MTF (40 ± 16%) compared to Anatase 16 (60 ± 10%). Moreover, the effluent toxicity (*Aliivibrio fischeri*) was reduced, and the degradation products were identified. Overall, TiO_2_ layers are a high-potential method for removing pharmaceuticals from MBR-treated greywater.

## Abstract 2

Immobilized TiO_2_ layers effectively treat greywater, enhancing pharmaceutical removal efficiency and reducing effluent toxicity for improved water safety.

## 1 Introduction

Access to safe and clean water is fundamental to sustainable development and public health, yet global industrialization and population growth have escalated the demand for freshwater, even for nonpotable uses. In this context, alternative water sources, such as greywater, emerge as viable solutions for reducing freshwater stress. Greywater, primarily consisting of domestic wastewater from sources like bathtubs, showers, and washbasins, presents a unique opportunity for recycling and reuse.^1^ However, emerging contaminants, particularly pharmaceuticals, pose significant challenges to their safe utilization.

The quest for effective greywater treatment has led to the extensive use of traditional methods such as membrane bioreactors (MBRs), filtration, adsorption, and reverse osmosis. These technologies are celebrated for producing high-quality effluent.^2−5^ Despite their effectiveness, these technologies exhibit limitations in removing pharmaceutical compounds, which resist conventional biological degradation due to their hydrophilicity and low molecular weight.^6^ Our preliminary studies have revealed considerable concentrations of pharmaceuticals such as naproxen (NPX), metformin (MTF), and sulfamethoxazole (SMX) in greywater effluents following MBR treatment, emphasizing the urgent need for supplementary treatment strategies.^7^

Several reports have been published on the successful degradation of pharmaceuticals by using powdered TiO_2_ photocatalysts. For example, Porcar-Santos et al.^8^ investigated the removal of SMX using TiO_2_ P25, achieving complete removal. Our previous study also obtained similar results for MTF and NPX.^7^ Other researchers have also reported comparable results.^9,10^ In all of these studies, powdered versions of TiO_2_ were used. Their use showed promise but had several drawbacks, including extra cost, energy, and technology expended in separating the powder from the treated effluent, making applying the technology complex.

To address this critical gap, our study introduces the novel application of immobilized TiO_2_ powders (Anatase 16, Anatase 5, and P25) on porous steel layers to efficiently degrade pharmaceuticals in greywater. These three TiO_2_ forms were chosen to assess variations in photocatalytic performance, as differences in particle size, surface area, and crystallinity can significantly affect the degradation efficiency. Anatase 16 and Anatase 5 vary primarily in particle size, while P25 is well-known for its mixed-phase composition, combining both anatase and rutile phases for enhanced photocatalytic activity. By comparing these forms, we aim to identify the most efficient variant for degrading pharmaceuticals in greywater. Additionally, this approach addresses the separation challenges of powdered photocatalysts and seeks to improve overall degradation efficiency. We characterized these TiO_2_ layers to understand their chemical–physical properties and assess their photocatalytic extensively, adsorptive, photolytic, and scavenging effects. Our investigation includes standard solutions of target pharmaceuticals and greywater samples subjected to preliminary MBR treatment. This study is distinctive in its integration of MBR technology with immobilized TiO_2_ layers for the targeted degradation of pharmaceuticals in greywater, marking a significant advancement in the field of water treatment and reuse.

## 2 Materials and Methods

### 2.1 Materials and Chemicals

Greywater was collected from buildings at Botanical, Praha 5, Czech Republic. All reagents used were of analytical grade. Sulfamethoxazole (SMX), metformin hydrochloride (MTF), and naproxen (NPX) were purchased from Sigma-Aldrich, Taufkirchen, Germany, while TiO_2_ (P25, Amorphous Anatase, and Anatase) plates were supplied by the J. Heyrovsky Institute of Physical Chemistry, Czech Academy of Science. Methanol, isopropyl alcohol, and sodium hydroxide were also of analytical grade from Penta, Czech Republic.

### 2.2 Preparation of Stock and Standard Solutions of the Pharmaceuticals

The stock solutions of NPX, MTF, and SMX were prepared by first producing 100 mg/L of pharmaceuticals in a standard 250 mL volumetric flask. The 100 mg/L concentration pharmaceuticals were then diluted 10 times to make another stock solution of 10 mg/L from which 500 μg/L was produced. However, in the production of SMX, the SMX was alkalized with 0.1 M NaOH to increase the solution’s solubility.

### 2.3 Quantitative Electrophoretic Deposition of TiO_2_ Nanoparticles

#### 2.3.1 Electrodeposition Protocol for TiO_2_ Nanoparticles

We used a controlled method for the electrodeposition of TiO_2_ nanoparticles to prepare the photocatalytic layers. Three types of TiO_2_ nanopowders, Anatase 16 (30 nm), Anatase 5 (14 nm), and P25, were utilized, dispersing each in 2-propanol (i-PrOH) at a concentration of 4 g/L. For the electrodeposition process, 0.5 mL of each suspension was mixed into 10 mL of specific organic solvents: a 2:3 ratio of i-PrOH and *n*-heptane for Anatase 16 and a 3:7 ratio of acetone and *n*-heptane for Anatase 5 and P25. Smooth silicon wafers were used as substrates due to their compatibility and suitability for photocatalytic testing.

The electrodeposition setup featured parallel stainless-steel plates as electrodes, each measuring 5 × 2.5 cm^2^, with a consistent distance of 1.1 cm between them. An electric field strength of 680 V/cm (750 V) was applied for 4 min, determined to be optimal for the complete deposition of TiO_2_ nanoparticles onto a 2.5 × 2.5 cm^2^ cathodic area (total deposition of 2 mg) with an area density of 0.3 mg·cm^–3^. This resulted in a TiO_2_ area density of 0.3 mg/cm^2^. The deposited layers exhibited porosity similar to the original nanopowders’ surface area.

#### 2.3.2 Characterization of Electrodeposited TiO_2_ Layers

The morphology of the electrodeposited TiO_2_ layers was examined by using scanning electron microscopy (SEM) to obtain high-resolution images of surface texture and particle size distribution. Samples were meticulously prepared to be contaminant-free. Transmission electron microscopy (TEM) was used for the detailed analysis of nanoparticle arrangement and crystalline structure, with careful dispersion on grids to maintain layer integrity. X-ray diffraction (XRD) characterized the crystalline structure, determining phase composition and confirming the presence of anatase TiO_2_. Chemical composition analysis using spectroscopic techniques verified the purity of TiO_2_ and the absence of impurities. Surface area and porosity measurements were conducted using gas adsorption techniques to quantify specific surface area and assess porosity, which is important for photocatalytic performance.

### 2.4 Photocatalytic Activity Assessment

**Figure 1:**
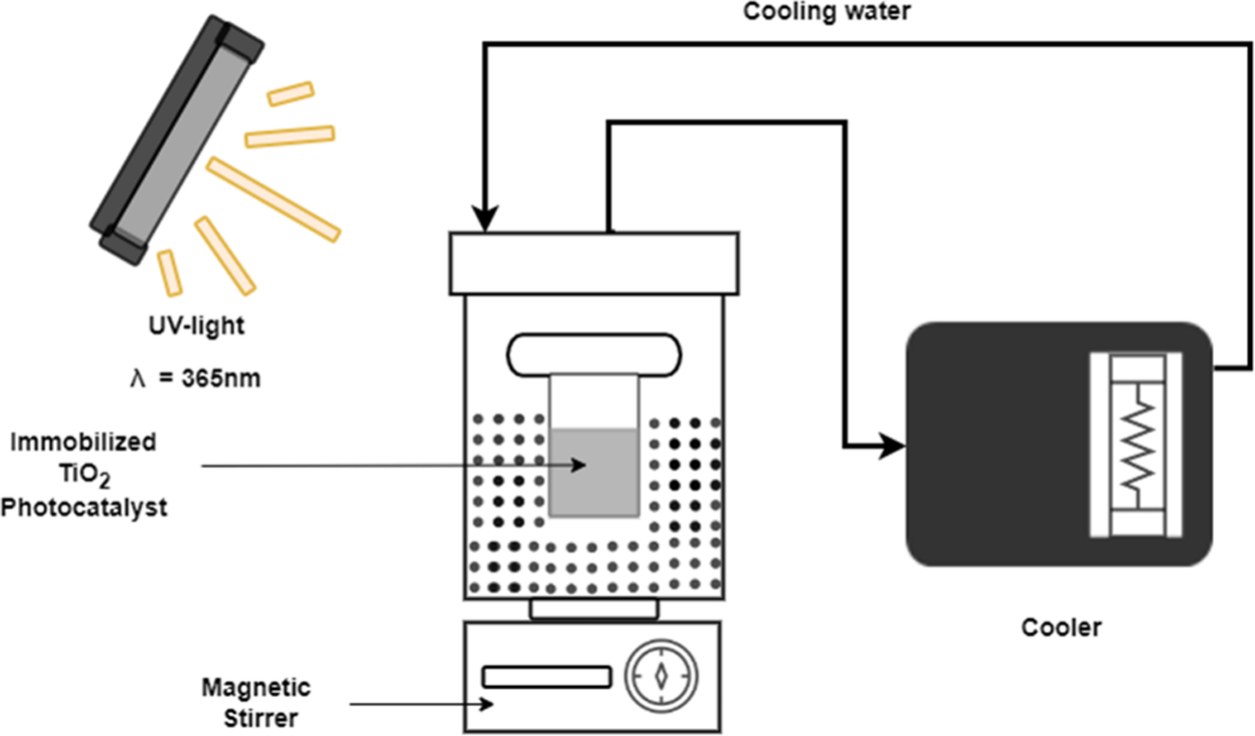
Photocatalysis experimental setup.

A bespoke bench-scale photocatalytic reactor was designed elaborately, focusing on the degradation efficacy of electrodeposited TiO_2_ layers against pharmaceutical compounds. Central to this setup was a UV-transparent, cylindrical chamber optimized to facilitate the effective UV-A irradiation essential for the TiO_2_ photocatalyst activation. The irradiation system (highlighted in Figure 1) comprised black-light fluorescence lamps (Philips TL-D BLB 15 W, a length of 45 cm, and a dominant wavelength of 365 nm), selected for their emission spectrum aligning with the TiO_2_ activation range. To ensure a uniform and consistent experimental condition, a radiometer (ILT 1400-A, International Light Technologies) with specific filters was employed to measure and monitor the light intensity and wavelength.

Initial tests were conducted with standard solutions of pharmaceuticals (NPX, SMX, and MTF) in ultrapure water, each at a concentration of 0.5 mg·L^–1^, followed by tests with greywater, spiked with the same concentration of pharmaceuticals (final concentrations were 0.52 ± 0.019, 0.54 ± 0.035, and 0.71 ± 0.027 for NPX, SMX, and MTF, respectively) and pretreated in a lab-scale MBR (concentrations were 0.43 ± 0.076, 0.42 ± 0.040, and 0.57 ± 0.053 for NPX, SMX, and MTF, respectively; Supporting Information Section 6 and Figure S4 outline the schematics of the lab-scale MBR treatment plants). The photocatalytical reactor’s operational integrity was maintained through meticulous cleaning of quartz cells after every use, and precise temperature regulation and increase of surface area were achieved using a cooling system (Julabo F12-ED Refrigerated and Heating Bath/Circulator) and an integrated stirrer (Velp Scientifica MULTISTIRRER15 Digital Magnetic Stirrer).

A critical aspect of this methodology was the sampling process during the photocatalytic experiments. UV-A light was introduced after a 10 min precontact period in darkness for adsorption and equilibration, marking the photocatalytic reaction’s beginning. At this stage, aliquots of 1 mL were systematically removed from the photocatalytic reactor at 1 h (60 min) intervals, continuing until the 300 min mark, and frozen at −20 °C before analysis. This regular sampling provided a detailed temporal profile of the photocatalytic degradation process.

The primary pharmaceuticals after treatment were detected by using UPLC-MS/MS using a 1290 Infinity II LC system (Agilent Technologies) with a 6460 triple quadrupole mass spectrometer (Agilent Technologies). The analysis parameters are outlined in Supporting Information Section 8, Tables S5 and S6.

For the photodegradation products, the UltiMate 3000 was coupled with a high-resolution tandem mass spectrometer Q-Exactive Plus (UHPLC–HRMS/MS; both Thermo Scientific, MA) equipped with an analytical column Acquity UPLC HSS T3 (100 × 2.1 mm^2^, 1.8 μm; Waters, MA) and electrospray (ESI) ionization interface: more information on the analysis is provided in Supporting Information Section 9, Table S7.

Unless explicitly stated otherwise, the kinetic analysis of the degradation of the pharmaceuticals followed first-order kinetics concerning bulking solutions, as indicated by the linear relationship in the plot of ln (*C*_0_/*C*) against time (*t*), enabling us to calculate the reaction rates (*k*).^11^ The removal efficiency of the process was computed as a percentage, reflecting the TiO_2_ layers’ capacity to reduce the concentration of the pharmaceuticals according to eqs 1–3
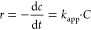
1
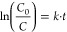
2
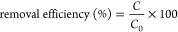
3where *r* = reaction rate, *t* = time (min), *k* = rate constant, *C* = concentration, and *C*_0_ = initial concentration. A plot of ln(*C*_0_/*C)* versus *t* provides *k*. The linearity of the plot proves the effectiveness of the fit.

### 2.5 Scavenging Test

The mechanism of photocatalytic degradation was investigated by conducting experiments with various reactive oxygen species (ROS) scavengers under the same photocatalytic conditions and solution concentrations as in previous tests: isopropanol (IPA) for hydroxyl radicals (OH^•^), sodium oxalate for holes (h^+^), sodium azide for singlet oxygen (^1^O_2_), and chloroform for superoxide radicals (O_2_^•–^). Selected for their specificity in quenching ROS, we facilitate the study of their roles in the degradation process.

### 2.6 Toxicity Assessment

The toxicity of the treated samples was examined; *Aliivibrio fischeri* and *Artemia salina* bioassays were employed. The procedures and results of these assessments are detailed in Supporting Information Section 7.

### 2.7 Reusability and Stability of TiO_2_ Layers

The stability and reuse potential of the electrodeposited TiO_2_ layers were assessed through multiple (10) photocatalytic cycles by using standard solutions of target pharmaceuticals. The experimental design, methods, and outcomes of these tests are described in Supporting Information Section 11.

### 2.8 Physicochemical Analysis

The greywater before and after treatment was meticulously assessed via physicochemical analytic methods. The detailed protocol encompassing the specific methodologies for sampling and subsequent analysis of the greywater before and after treatment is elaborately presented in Supporting Information Section 6.

### 2.9 Photocatalytic Treatment of Greywater

The efficacy of the TiO_2_ layers in treating greywater post-MBR processing was evaluated. First, the raw greywater was spiked with 0.5 mg·L^–1^ of each pharmaceutical and treated using the lab-scale MBR plant. Samples were collected and treated with TiO_2_ layers following the same protocol as for standard solutions. The samples were then stored and analyzed as done with the standard solutions. Ten runs were conducted for each plate.

## 3 Results

### 3.1 Comparative Analysis of Physicochemical Properties of Immobilized Porous TiO_2_ Layers

**Figure 2:**
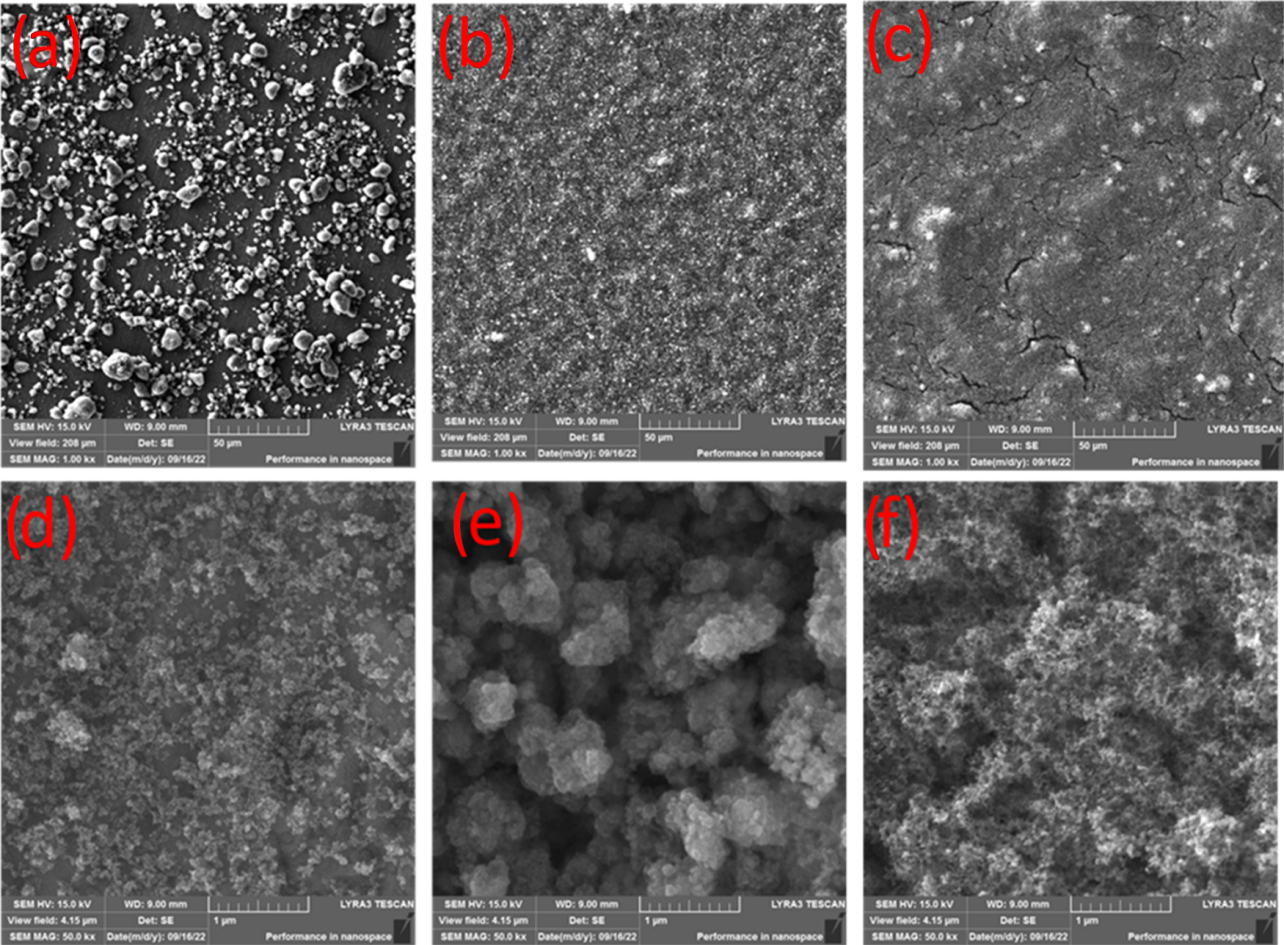
SEM images of the immobilized plate: first column Anatase 16, Anatase 5, third column P25 (note that the first and second rows are for the 50 μm and 1 μm scales, respectively).

This study examined the physical and chemical properties of the three immobilized TiO_2_ layers—Anatase 16, Anatase 5, and P25, used in the photocatalytic degradation of target pharmaceuticals in deionized water and MBR-treated greywater. SEM analysis (Figure 2) revealed better adhesion properties for Anatase 5 and P25 than Anatase 16, with observed cracks in P25 layers due to drying effects.^12^ 3D surface studies (Figure S1) showed P25 layers to have more consistent and uniform surfaces due to particle aggregation, affecting surface roughness but not functionality.

XRD analysis (Figure S2) confirmed the presence of anatase, rutile, and potential brookite phases in the layers, indicating a complex crystalline structure influenced by milling, which could affect the photocatalytic properties. FT-IR spectroscopy (Figure S3) identified strong CO absorption, indicative of the presence of CO-Ti^4+^ complexes on the TiO_2_ surface, crucial for photocatalytic activity. Comparative physical assessments, as shown in Table S1, revealed that Anatase 5 has the highest specific surface area, at 115 m^2^·g^–1^. In contrast, Anatase 16 and P25 surface areas of 48 and 50 m^2^·g^–1^, respectively, are less than half of that of Anatase 5. All samples demonstrated similar stability and band-gap energies, indicating comparable photocatalytic activation requirements.

### 3.2 Photocatalytic Performance of the TiO_2_ Layers in Standard Solutions

**Figure 3:**
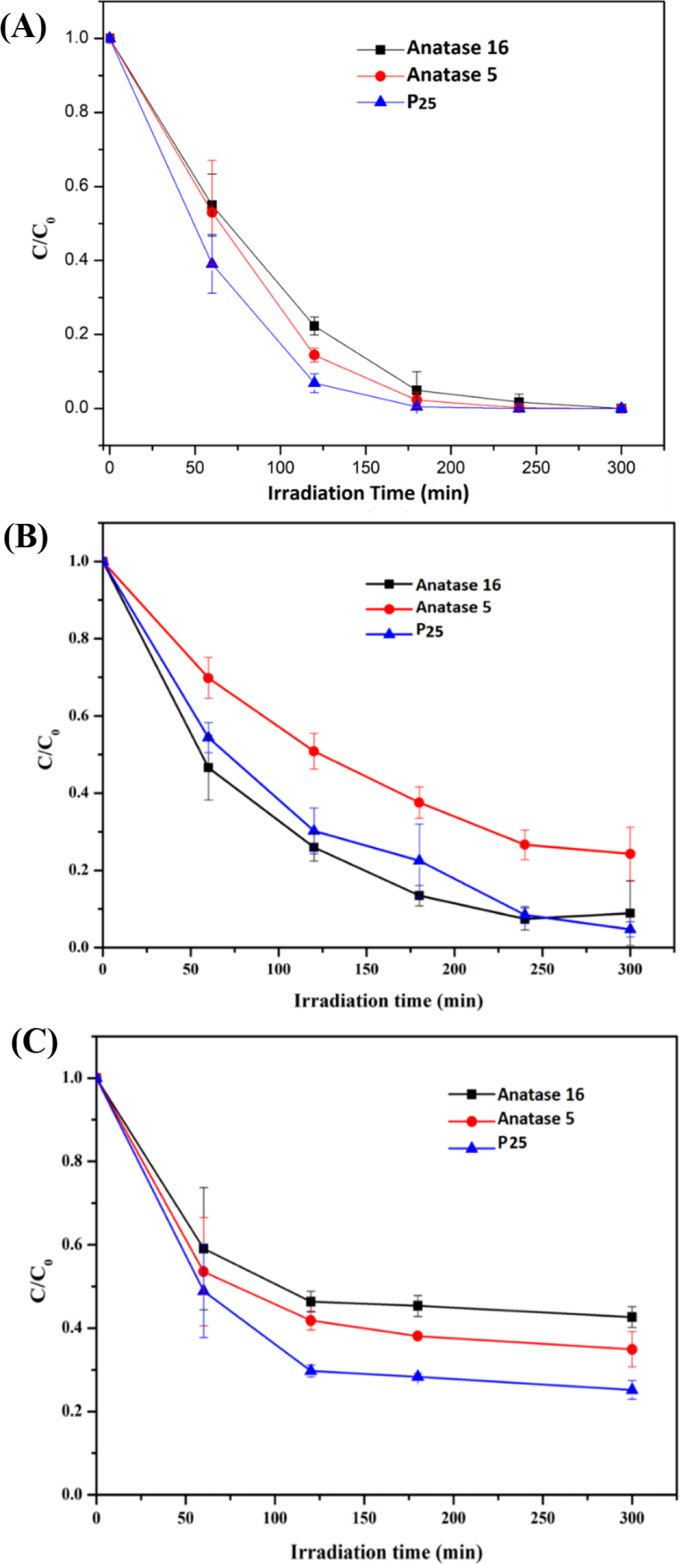
Photocatalytic degradation of NPX (A), SMX (B), and MTF (C) in standard solution using Anatase 16, Anatase 5, and P25 layers conducted in triplicate.

Figure 3 offers a detailed visual representation of the time-dependent photocatalytic degradation of three pharmaceuticals (NPX, SMX, and MTF) using Anatase 16, Anatase 5, and P25 layers. Detailed quantifications of these compounds’ degradation percentages and regression analyses are thoroughly detailed in the Supporting Information in Table S2.

Furthermore, the detection methodologies for these analytes and their TPs using UPLC-MS/MS and UHPLC-HRMS/MS are detailed in Supporting Information Sections 8 and 9, respectively.

#### 3.2.1 NPX Degradation Efficiency

Remarkably, the photocatalytic process employed using all three types of layers resulted in the effective degradation of NPX. Notably, after 300 min, we observed the complete removal of NPX from the standard solution. The degradation kinetics, following a pseudo-first-order model, yielded rate constants of 0.018 min^–1^ for P25, 0.014 min^–1^ for Anatase 5, and 0.012 min^–1^ for Anatase 16, demonstrating a nuanced variance in the efficiency of these photocatalysts.

In reusability studies (Figure S7A), degradation remained consistently high across three cycles for each TiO_2_ layer, achieving a 100% removal of NPX after 300 min, highlighting the layers’ stability and reusability.

TP studies on NPX degradation (Table S8) revealed that the process involves initial hydroxylation, introducing hydroxyl groups into the aromatic ring or naphthalene structure, evidenced by TPs such as TP1, TP4, TP5, and TP7 as highlighted in Figure S5. Subsequent ring opening, indicated by TP2 and TP3, signifies further breakdown into simpler forms, highlighting significant alterations in the NPX molecule. Additionally, decarboxylation, suggested by TP6, represents the removal of carboxyl groups, leading to smaller aromatic compounds.

#### 3.2.2 SMX Degradation Efficiency

For SMX degradation, Anatase 16 TiO_2_ emerged as the most effective, exhibiting the highest rate constant of 0.012 min^–1^. P25 and Anatase 5 followed, with degradation rate constants of 0.010 and 0.005 min^–1^, respectively. Notably, Anatase 5′s lower degradation rate, approximately half that of Anatase 16, led to incomplete degradation of SMX after 300 min. The degradation efficiencies observed were 95% for P25, 91% for Anatase 16, and 76% for Anatase 5, underlining the outstanding performance of both Anatase 16 and P25 in this context.

Reusability studies (highlighted in Figure S7B) showed different efficiencies among the TiO_2_ layers; Anatase 16 maintained relatively high degradation rates, slightly decreasing from 97 to 90% over three cycles. In contrast, Anatase 5 experienced a more significant drop in efficiency, from 83 to 69%, while P25 showed minor reductions, maintaining above 90% efficiency throughout.

SMX degradation (Table S8) is characterized initially by the cleavage of the S–N bond, crucial for the compound’s breakdown, as shown by the formation of TP2 and TP3. Modifications to the sulfonate group, observed in TPs such as TP1, TP5, and TP6, indicate oxidative changes affecting the sulfonamide group, signifying oxidative degradation processes (Figure S6). Furthermore, detecting a high-molecular-weight TP7 suggests potential dimerization or polymerization, forming more complex compounds from other transformation products.

#### 3.2.3 MTF Degradation Analysis

**Figure 4:**
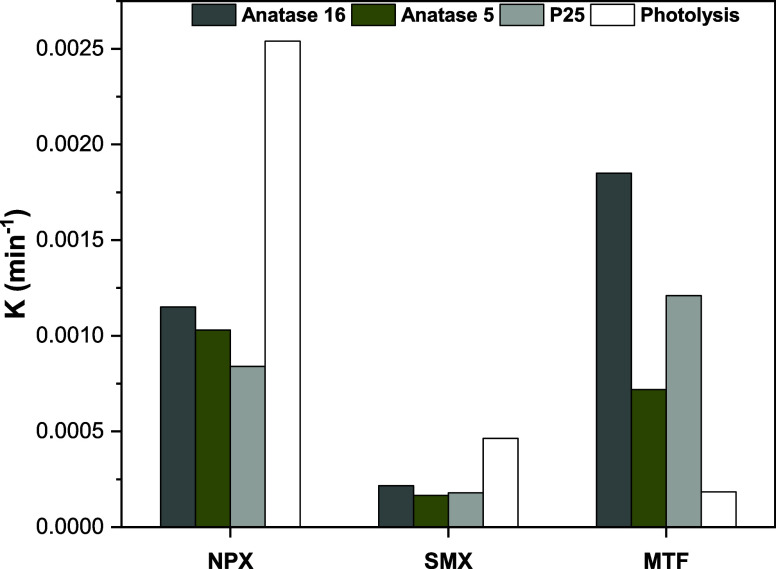
Adsorption and photolysis reaction rates of Anatase 16, Anatase 5, and P25 for each target pharmaceutical.

MTF degradation was analyzed using pseudo-first-order kinetics to determine the rate constants of the photocatalytic reactions. P25 exhibited the highest degradation efficiency and percentage degradation, clocking in at 0.009 min^–1^ and 75%, respectively. In contrast, Anatase 16 and Anatase 5 exhibited slower reaction rates, approximately half that of P25, at 0.005 and 0.006 min^–1^, as depicted in Figure 4. The overall degradation efficiency of these layers was 57 and 65%, respectively, after 300 min. A plateau in MTF degradation was observed across all photocatalysts, possibly attributable to the saturation of active degradation sites on the TiO_2_ surface. This phenomenon could be further exacerbated by TPs such as guanyl urea, which might compete for the available photocatalytic sites. Similarly, during recyclability studies (Figure S7C), MTF degradation presented lower efficiencies overall, with a noticeable decline over successive cycles; Anatase 16’s efficiency decreased from 64 to 43%, Anatase 5 from 74 to 32%, and P25 from 73 to 54%, indicating varying degrees of photostability and photocatalyst deactivation, possibly due to the accumulation of intermediate degradation products.

MTF degradation begins with deamination, removing amine groups from the molecule, as seen in TP1 and TP2 (Table S8). This step is pivotal for the initial breakdown. Oxidation reactions leading to the formation of TP3 and TP4 indicate modifications to the methyl group or the imidazole ring, further altering MTF’s chemical structure (Figure S7).

### 3.3 Photolysis of Target Pharmaceuticals

Our study extended our analysis to include NPX, SMX, and MTF photodegradation with the same initial concentrations in standard solutions, using solely UV-A irradiation without the photocatalytic layers (reaction rates illustrated in Figure 4). After 300 min of exposure, the degradation efficiencies were observed to be 50% for NPX, 13% for SMX, and 7% for MTF. Notably, NPX exhibited the highest susceptibility to photodegradation under UV-A light, while SMX and MTF demonstrated considerable photostability, indicating inherent differences in their molecular structures affecting their responsiveness to UV-A irradiation.

### 3.4 Adsorption of Pharmaceuticals on TiO_2_ Layers

This study also examined NPX, SMX, and MTF adsorption behaviors on TiO_2_ layers in standard solutions (see Figure 4). For NPX, adsorption equilibrium was reached within 180 min for Anatase 16 and Anatase 5, achieving removal efficiencies of 42 and 27%, respectively. The adsorption capacities for these layers were calculated as 0.12 mg·g^–1^ for Anatase 16 and 0.18 mg·g^–1^ for Anatase 5. Interestingly, P25 required a longer duration of 300 min to reach adsorption equilibrium, resulting in removal efficiencies of 21, 20, and 30% for Anatase 16, Anatase 5, and P25, respectively, with P25 demonstrating an adsorption capacity of 0.18 mg·g^–1^.

The adsorption dynamics for SMX revealed that equilibrium was attained at 180 min, with respective removal efficiencies of 8, 4, and 6% for Anatase 16, Anatase 5, and P25 (Figure 4). The corresponding adsorption capacities were 0.46 mg·g^–1^ for Anatase 16, 0.48 mg·g^–1^ for Anatase 5, and 0.47 mg·g^–1^ for P25, suggesting a relatively uniform adsorption potential across the different TiO_2_ layers.

Similarly, for MTF, the adsorption equilibrium was achieved at 180 min. The removal efficiencies were approximately 42% for Anatase 16 and 26% for Anatase 5 and P25. The adsorption capacities at this equilibrium point were 0.29 mg·g^–1^ for Anatase 16 and 0.37 mg·g^–1^ for Anatase 5 and P25, indicating a distinct adsorption behavior for MTF compared to that of NPX and SMX.

### 3.5 Mechanism Inferred from Scavenging Tests

**Figure 5:**
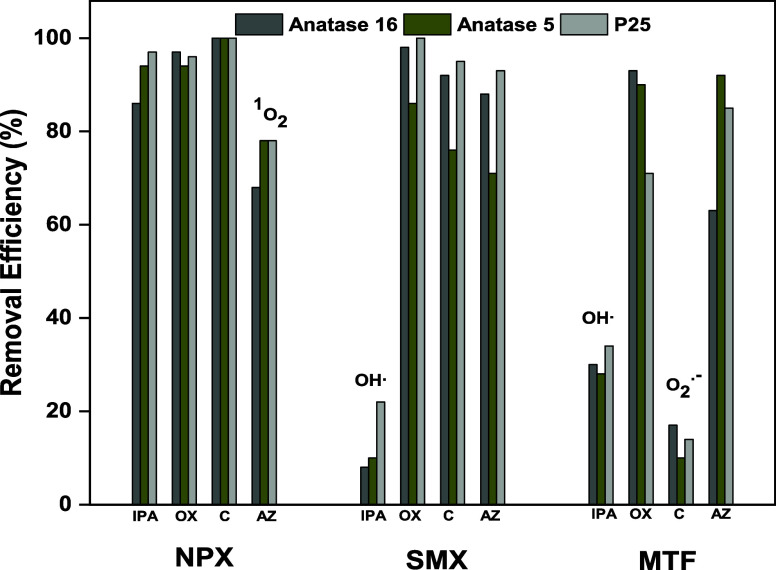
Effect of scavengers on the degradation of target pharmaceuticals (NPX, SMX, and MTF) after 300 min of irradiation. IPA, isopropyl alcohol 0.1 M; OX, sodium oxalate 1 mM; C, chloroform 0.1 M; and AZ, sodium azide 1 mM. ROS scavenged by the most efficient scavengers is highlighted in the figure.

The degradation efficiencies of NPX, SMX, and MTF were significantly impacted by the presence of ROS and their scavenging, as shown in Figure 5. For NPX, adding IPA to quench OH^•^ radicals slightly reduced degradation efficiencies to 86, 94, and 97% for Anatase 16, Anatase 5, and P25, respectively, underscoring the importance of OH^•^ in the degradation process. Quenching ^1^O_2_ more markedly decreased NPX degradation to 68, 78, and 78%, respectively, highlighting ^1^O_2_’s critical role. For SMX, scavenging OH• with IPA dramatically decreased degradation efficiencies to 7.7, 10.5, and 22.0%, indicating OH^•^’s dominant role in its degradation. Conversely, scavenging other ROS such as O_2_^•–^, h^+^, and ^1^O_2_ had varying impacts, with significant effects observed for Anatase 16 and P25 but a minimal effect on Anatase 5. MTF degradation was considerably affected by the quenching of ^1^O_2_ using chloroform, resulting in degradation efficiencies of 17, 10, and 14% for Anatase 16, Anatase 5, and P25, respectively. Similarly, scavenging OH^•^ also reduced MTF degradation efficiencies to 30, 29, and 34%, respectively.

### 3.6 MBR Treatment of Greywater with Pharmaceutical Residues

Over a 3 month study, the MBR treatment process (setup highlighted in Figure S4) significantly improved the quality of greywater, as detailed in Table S3. Conductivity was reduced slightly from 0.00028 to 0.38 to 0.002 to 0.29 mS·cm^–1^ post-treatment, indicating some removal of dissolved ions. pH levels remained stable within the optimal range of 7.0–8.3 for raw and 7.2–8.0 for treated greywater, supporting its suitability for various applications. Total suspended solids (TSS) were eliminated to nondetectable levels from an initial range of 14.5–115.0 mg·L^–1^, showcasing the MBR’s clarifying capability, although total dissolved solids (TDS) saw a slight adjustment from 227–327 mg·L^–1^ in raw to 240–402 mg·L^–1^ in treated water, indicating changes in dissolved constituents.

COD significantly decreased, with soluble COD (sCOD) dropping from 12–73 to 1–25 mg·L^–1^ and total COD (tCOD) from 51–670 to 1–36 mg·L^–1^, reflecting organic matter and pollutant reduction. Nutrient levels of ammonia, nitrite, and nitrate were also substantially reduced, enhancing the treatment effectiveness. Remarkably, the treated greywater met EU Reuse and Drinking Water Directives’ standards, with conductivity, TSS absence, reduced COD, and nutrient levels within acceptable limits for reuse and irrigation. This compliance underscores the treated greywater’s potential for sustainable applications, emphasizing the MBR process’s efficacy.

Afterward, the collected greywater was spiked to a concentration of 0.5 mg/L, followed by tests with greywater spiked with the same concentration of pharmaceuticals, resulting in final concentrations of 0.52 ± 0.019 mg·L^–1^ for NPX, 0.54 ± 0.035 mg·L^–1^ for SMX, and 0.71 ± 0.027 mg·L^–1^ for MTF. After pretreatment in a lab-scale MBR, the concentrations were reduced to 0.43 ± 0.076 mg·L^–1^ for NPX, 0.42 ± 0.040 mg·L^–1^ for SMX, and 0.57 ± 0.053 mg·L^–1^ for MTF. While the MBR treatment demonstrated reductions in pharmaceutical concentrations—17.3% for NPX, 22.2% for SMX, and 19.7% for MTF—the removal rates are relatively modest. Although these reductions indicate some level of pharmaceutical removal, they suggest that additional treatment or optimization may be required for more effective mitigation of these micropollutants in greywater.

### 3.7 MBR-TiO_2_ Treatment of Greywater for Pharmaceutical Degradation

**Figure 6:**
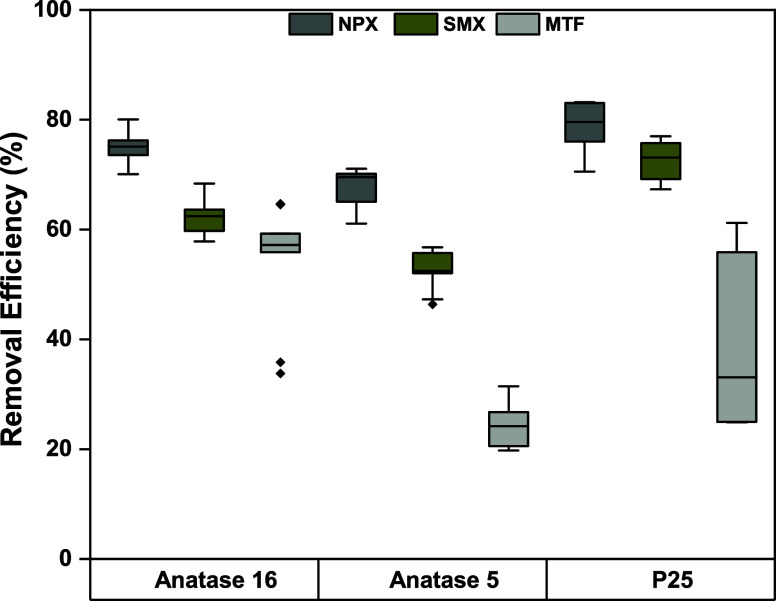
Degradation of mixed pharmaceuticals in greywater by Anatase 16, Anatase 5, and P25, respectively. Ten cycles were run for each photocatalyst. The lower and upper limits of the boxes represent the 25th and 75th percentiles, respectively. The middle line within the box corresponds to the median. The whiskers of the plot extend to 1.5 times the interquartile range, and the yellow diamond indicates outlier values defined as data points that fall more than 1.5 times the interquartile range away from the upper or lower quartiles.

Evaluating the degradation efficiency of a mixture of pharmaceuticals in MBR-treated greywater using different TiO_2_ layers was conducted. The MBR treatment was conducted, and the analyzed removal efficiencies are highlighted in Figure 6. This assessment aimed to understand these photocatalysts’ real-world applicability, reusability, and durability across 10 runs. P25 layers excelled, particularly against NPX and SMX, with removal efficiencies of 78 ± 5% and 73 ± 4%, respectively, though less effective for MTF at 40 ± 16%. Anatase 16 showed strong performance, especially with NPX at a 75 ± 3.4% removal rate, and moderate success with SMX and MTF at 64 ± 3.8% and 60 ± 10%, respectively. Meanwhile, Anatase 5 layers achieved decent removal rates for NPX and SMX at 67 ± 3.9% and 53 ± 3.7%, respectively, but struggled with MTF, only reaching a 24 ± 3.9% efficiency.

The study’s toxicity assessment on greywater (highlighted in Supporting Information Section 7), using both MBR and subsequent MBR-TiO_2_ treatments, showed no mortality in *Artemia salina*, indicating nonacute toxicity. Evaluations with *Aliivibrio fischeri* revealed high toxicity in untreated greywater, significantly reducing bioluminescence. Post-MBR treatment, toxicity notably decreased, and this reduction was further enhanced by the MBR-TiO_2_ process (Table S4).

## 4 Discussion

### 4.1 Morphological and Chemical Characterization of TiO_2_ Photocatalysis

The morphological and chemical attributes of Anatase 16, Anatase 5, and P25 TiO_2_ photocatalysts highlight their capabilities and limitations for degrading pharmaceuticals in MBR-treated greywater. Anatase 5 and P25 showed superior substrate adherence compared to Anatase 16, impacting photocatalytic stability and effectiveness.^13,14^ P25’s cracks, linked to particle size and drying behavior, could compromise its stability, although its uniform surface and pronounced aggregation enhance photocatalytic performance.^15,16^

XRD and FT-IR analyses confirmed complex crystalline structures and reactive surface chemistry, with anatase and rutile phases in P25 indicating a reactive interface.^17^ The absence of Ti–OH groups suggests tailored chemical properties that influence photocatalytic reactions. Anatase 5′s higher surface area offers more active sites for degradation, potentially increasing pharmaceutical removal efficiency. Similar ζ-potential values indicate uniform dispersion stability, while aligned band-gap energies suggest a similar energetic threshold for initiating photocatalytic reactions.^18^

### 4.2 Quantitative Analysis of Photocatalytic Degradation Efficiency Using TiO_2_ Layers

In assessing TiO_2_ photocatalysts for pharmaceutical degradation, P25 emerged superior in standard solution trials, particularly for MTF degradation. This efficiency is attributed to the 80:20 anatase-to-rutile composition, leveraging anatase’s high photoactivity and rutile’s substantial adsorption capacity to minimize electron–hole pair recombination.^7,19^ Although anatase’s particle size benefits photoactivity, its tendency to agglomerate lowers its surface area and adsorption potential compared to rutile.^7^ This balance enhances ROS production and retention, underpinning P25’s superior degradation capabilities.

For NPX degradation, all TiO_2_ layers achieved complete breakdown due to NPX’s UV-A light absorption, increasing its photocatalytic susceptibility. P25’s anatase–rutile mix promoted photoactivity and adsorption, with O_2_^•–^ identified as the predominant ROS, contrary to the literature emphasizing OH^•^.^20,21^ NPX photodegradation pathways include decarboxylation and hydrogen abstraction, leading to intermediates like 2H-1-benzopyran-2-one, further breaking down into stable products.^22,23^

Despite the complex structure of SMX, P25 demonstrated unparalleled degradation capabilities due to its augmented photocatalytic activity and efficient interfacial electron transfer.^24^ Minimal adsorption of SMX to TiO_2_ suggests optimization potential to enhance degradation efficiency. OH^•^ and electron–hole pairs play pivotal roles in SMX degradation, producing seven transformation products, including hydroxylated variants and derivatives like sulfanilic acid and aniline.^25,26^

MTF degradation posed unique challenges, with P25 showing a reduced efficiency due to insufficient ROS production. Incorporating electron acceptors into the TiO_2_ matrix may enhance MTF degradation efficiencies.^27^ MTF degradation pathways involve OH^•^ radicals, leading to demethylation and formation of transformation products such as methyl biguanide and guanyl urea.^28−30^ The recyclability studies conducted using the plate demonstrate the enduring efficacy of TiO_2_ layers in removing NPX and SMX, showing high removal efficiency through successive treatments highlighting their potential for greywater treatment. Anatase 16 and P25 effectively eliminated MTF, with P25’s rutile-anatase blend and Anatase 16’s superior crystallinity-enhancing performance.^31^ Conversely, Anatase 5′s performance declined due to the accelerated recombination of photogenerated electron–hole pairs and surface traps.^31−33^ Introducing thin, porous photocatalytic plates may improve reusability but requires balancing mechanical durability and catalytic performance.^34,35^

This study also provides valuable insights into the crucial roles of ROS in degrading NPX, SMX, and MTF during photocatalysis while highlighting the complex dynamics of ROS with immobilized porous plates. The structural properties of TiO2 photocatalysts, especially P25’s anatase-to-rutile ratio, enhance ROS generation and retention, making them highly effective for degrading pharmaceutical contaminants. Additionally, the incorporation of thin, porous photocatalytic plates increases reusability by expanding active sites. The TiO_2_ layers demonstrated excellent stability, as they could be reused up to 10 times (a total of 50 h for each layer) with almost constant efficacy (Figure S7).

### 4.3 Adsorption Behavior and Its Impact on Photocatalytic Degradation

Our study explored the adsorptive behaviors of NPX, SMX, and MTF on TiO_2_ photocatalysts, highlighting the impact of adsorption processes on the photocatalytic degradation of these pharmaceuticals. We carefully examined the ζ-potentials of the photocatalysts and their interactions with the pollutants, providing insights into how adsorption dynamics influence photocatalysis.

The analysis of ζ-potentials for the three TiO_2_ photocatalysts in demineralized water and greywater revealed predominantly negative surface charges consistent across different water matrices. Interestingly, dissolved species in greywater did not significantly alter the TiO_2_ particles’ surface charge, indicating minimal impact on the photocatalyst’s adsorptive properties in varying aqueous environments.

For NPX, we observed moderate adsorption levels on the TiO_2_ plates, aligning with other studies that suggest that pH conditions (maintained at 7) lead to ionic interactions forming relatively weak intermolecular bonds between NPX and TiO_2_.^36,37^ Such weak bonding may facilitate the re-entry of NPX into the aqueous phase, as noted for Anatase 16 and Anatase 5. However, P25 exhibited a notable exception, demonstrating higher removal efficiency and suggesting stronger intermolecular bonds that more effectively retain NPX on the photocatalyst’s surface over time.

SMX showed the least effective adsorption on all TiO_2_ layers, attributed to its nonpolar nature and pH-dependent charge distribution. The disparity in polarity between SMX and the highly polar TiO_2_ and the repulsive interactions between their negative charges at pH 7 led to significantly reduced adsorption.^38^ These findings underscore the importance of photocatalytic degradation as a more effective strategy for removing SMX from wastewater as opposed to adsorption-based methods, which may not fully decompose the pollutant.

The adsorption of MTF on TiO_2_ surfaces was moderate and influenced by the drug’s p*K*_a_ values and the resulting charge form at different pH levels. At the pH used in this study, MTF predominantly existed in a monoprotonated form, which may hinder its electron-accepting ability and its interaction with the photocatalyst. This reduced interaction potentially led to lower adsorption capacities, highlighting the complexities of adsorption phenomena for different pharmaceuticals under varying pH conditions.^39^

### 4.4 Photostability of Target Pharmaceuticals in Water Treatment Processes

This study meticulously explored the photostability of NPX, SMX, and MTF under varying light exposure conditions, providing insightful observations on their behavior in photocatalytic processes.

NPX showcased a distinctive photostability profile that can absorb light within the 315–400 nm range, aligning with the emission spectrum of the commonly used UV lamps, particularly at 365 nm. This spectral compatibility facilitated NPX’s photodegradation, primarily through light absorption that leads to excited electronic states and subsequent decomposition reactions, including rearrangement, isomerization, and ionization.^40^ Additionally, the formation of OH• during the photolysis process and the interaction of oxygen in open systems contributed to NPX decomposition, further aided by processes like photocarboxylation. Integrating TiO_2_ photocatalysts significantly enhanced NPX removal efficiency, offering a robust solution for eliminating this pharmaceutical from water systems.^22^

SMX exhibited a peak UV-A absorbance at 262 nm, making it susceptible to direct photolysis degradation, particularly targeting dissociable bonds such as –NH_2_, –S–, –NH–, and −N–O–.^41^ However, direct UV photolysis at 365 nm achieved limited success due to SMX’s minimal absorption at this wavelength. While direct photolysis presented a somewhat effective removal method, incorporating TiO_2_ under UV-A light at 360 nm significantly increased SMX’s degradation efficiency, underlining the superior effectiveness of photocatalysis over simple photolysis in SMX removal.^42^

MTF exhibited the lowest direct photodegradation efficiency under UV-A light, with only a 9% removal efficiency observed after 300 min. This suggested that MTF likely does not absorb light at 360 nm effectively, implying that direct photolysis with UV-A light of lower wavelengths might be more conducive to MTF degradation. This characteristic points to the need to optimize the wavelength of UV light used in the photolysis process to enhance MTF degradation efficiency or explore alternative photocatalytic methods for its effective removal.^43^

The investigation into the photostability and degradation pathways of NPX, SMX, and MTF highlighted the critical role of light absorption characteristics and the molecular structure of pharmaceuticals in determining their degradation efficiency in photocatalytic processes. The findings emphasize the importance of selecting appropriate wavelengths for UV photolysis and the enhanced degradation capabilities offered by TiO_2_ photocatalysis, showcasing the potential for tailored approaches in the photodegradation of pharmaceutical pollutants.

### 4.5 MBR-TiO_2_ Treatment of Graywater

When effluent from MBR was treated using the trio of photocatalysts, a marginal decline in the pharmaceutical degradation efficacy was observed. This reduction is attributed to the presence of dissolved organic matter (DOM) in the effluent, which competes for ROS or functions as ROS scavengers, diminishing the photocatalytic process’s effectiveness.^44,45^ Despite this, NPX and SMX were efficiently degraded by all three photocatalysts, demonstrating the viability of this technology for purifying greywater contaminated with these pharmaceuticals. Their analogous molecular structures, sharing similar functional groups and molecular weights, likely facilitate comparable degradation efficiencies through the action of hydroxyl radicals (OH^•^).^28^

Conversely, MTF exhibited variable removal efficiencies and greater resistance to degradation compared to those of NPX and SMX across all photocatalytic plates. This resilience is likely due to the absence of aromatic rings in MTF’s molecular structure, necessitating higher energy input or distinct reaction conditions for effective degradation.^46^ Factors such as the initial concentration of MTF, solution pH, and light source intensity could further influence the effectiveness.^47^ Among the photocatalysts, Anatase 16 showed a higher MTF removal efficiency than P25 and Anatase 5 TiO_2_, attributed to its larger surface area and more active sites. Conversely, Anatase 5 TiO_2_ exhibited the lowest removal efficiency for MTF due to its smaller particle size and reduced surface area.^34^

The synergy of MBR technology and photocatalysis offers a promising approach to reducing greywater toxicity by completely mineralizing pharmaceutical residues. This study emphasizes the importance of complete mineralization to effectively diminish toxicity, as partially degraded transformation products can still contribute to bioassay toxicity.^48^

Elongating the reaction time in the photocatalysis segment of the MBR-photocatalysis treatment can enhance mineralization, particularly with Anatase 16 and Anatase 5 photocatalysts, suggesting that the prolonged TiO_2_ interaction improves pollutant degradation. The complex toxicity dynamics of greywater, composed of various chemicals and their transformation products, underscore the need for complete mineralization to reduce both individual and collective risks.^49,50^ This approach mitigates toxicity and enhances the safety of treated greywater for reuse applications.^51^ Advancing greywater treatment technologies have significant environmental and public health implications.

## 5 Conclusions

This investigation has illuminated the promising capabilities of immobilized TiO_2_ layers in purifying MBR-treated greywater from pharmaceutical contaminants. The photocatalytic prowess of three distinct TiO_2_ variants (P25, Anatase 16, and Anatase 5) was scrutinized under UV-A illumination, focusing on removing NPX, SMX, and MTF—pharmaceuticals frequently detected in environmental waters. The outcomes were revealing; all tested TiO_2_ layers demonstrated significant efficacy in decontaminating the water, showcasing complete elimination of NPX and notable degradation rates for SMX and MTF. Among these, P25 TiO_2_ emerged as the most potent, achieving superior degradation reaction rates and efficiencies, as well as toxicity reduction, across standard solutions and MBR-treated greywater. This study also expanded our understanding of pharmaceutical degradation pathways, revealing that OH^•^ was not the only reactive species involved. Other ROS, such as h^+^, ^1^O_2_, and O_2_^•–^, also played significant roles. Multiple intermediaries were identified, highlighting the complexity of the degradation process. Additionally, the research validated the reusability of TiO_2_ layers, proposing a sustainable approach for prolonged water treatment use.

## Supporting Information Available

Additional experimental details, materials and methods, including figures for TiO_2_ layer characterization (e.g., SEM, XRD), degradation kinetics, and toxicity data (PDF)
