# Supplementary material for: Efficient Degradation of Recalcitrant Pharmaceuticals in Greywater Using Treatment of MBR and Immobilized TiO2 Porous Layers
Source: ACS ES T Water. 2024 Nov 29;4(12):5587–97. doi: 10.1021/acsestwater.4c00618 (PMC11650637; doi:10.1021/acsestwater.4c00618)
Supplement: Supplementary file 1 — ew4c00618_si_001.pdf [file ew4c00618_si_001.pdf]

SUPPLEMENTARY INFORMATION

Efficient degradation of recalcitrant pharmaceuticals in greywater using treatment of MBR and immobilized TiO_2 porous layers

Bukola Ojobe[a], Idris Okeowo[a], Jiri Rathousky[b], Libor Brabec[b], Tereza Marikova[b,c], Eliska Mikyskova[b], Jana Kofronova[d], Radek Vurm[d], Stepanka Smrckova[a], Saeed Jamali Ashtiani[e], Karel Friess[e], Zbynek Dzuman[f], Vojtech Kouba[a*] , Jan Bartacek[a]

a. Department of Water Technology and Environmental Engineering, University of Chemistry and Technology Prague, Technicka 5, 166 28 Prague, Czech Republic
b. Center for Innovations in the Field of Nanomaterials and Nanotechnologies, J. Heyrovsky Institute of Physical Chemistry, Czech Academy of Sciences, Dolejskova 3, 182 23 Prague, Czech Republic
c. Forensic Laboratory of Biologically Active Substances, Department of Chemistry of Natural Compounds, University of Chemistry and Technology Prague, Technicka 5, 166 28 Prague, Czech Republic
d. Department of Environmental Chemistry, University of Chemistry and Technology Prague, Technická 5, 166 28 Prague, Czech Republic
e. Department of Physical Chemistry, University of Chemistry and Technology, Technická 5, 166 28 Prague, Czech Republic
f. Department of Food Analysis and Nutrition, University of Chemistry and Technology, Technická 5, 166 28 Prague, Czech Republic

* Corresponding author koubav@vscht.cz,

1. 3D surface images of the TiO_2 Layers

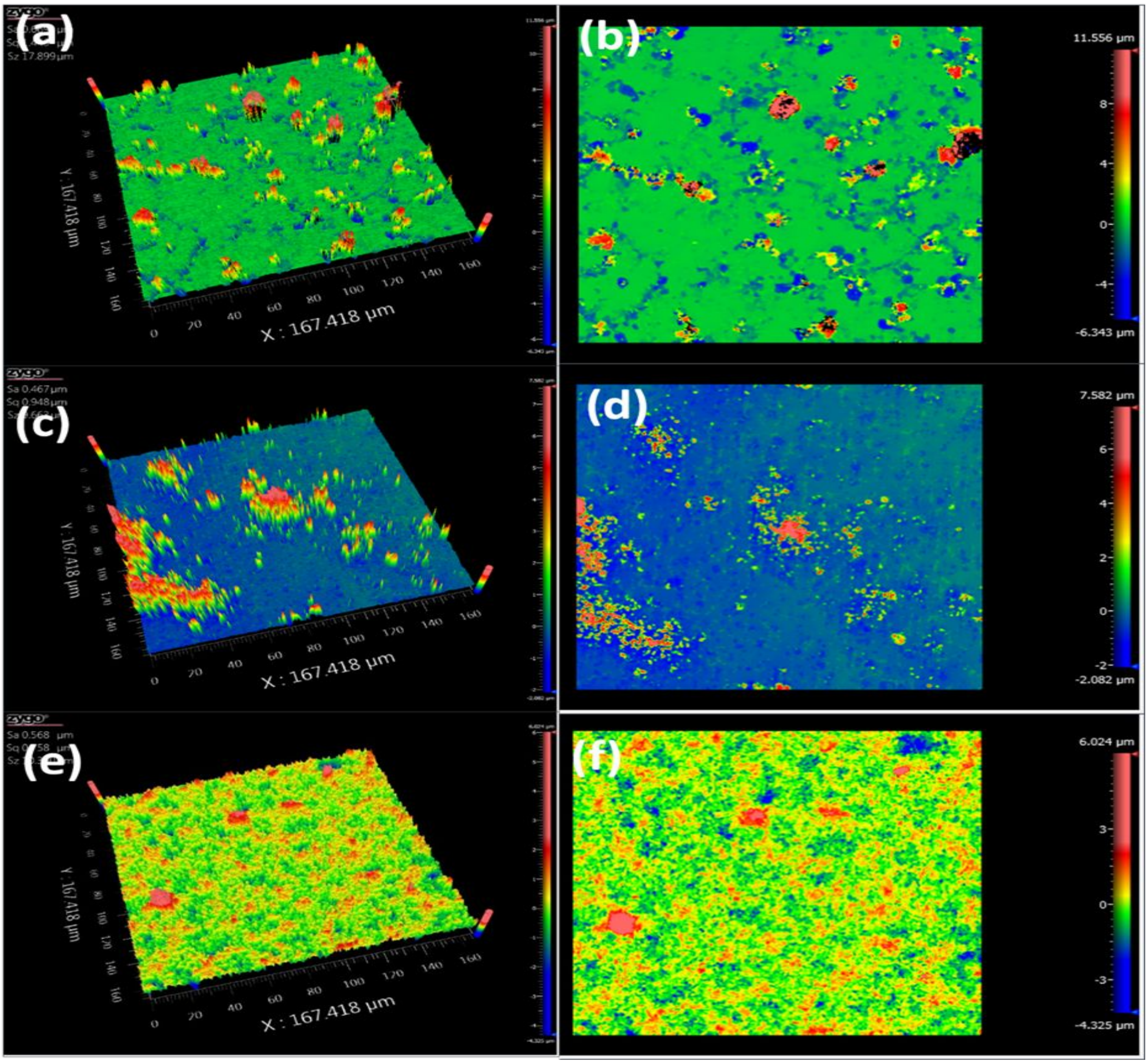

Figure S1. The 3D images of the tested samples containing different TiO_2 crystals: 1st row Anatase 16, 2nd row Anatase 5, 3rd row P25.

2. XRD Analysis of the TiO_2 Layers

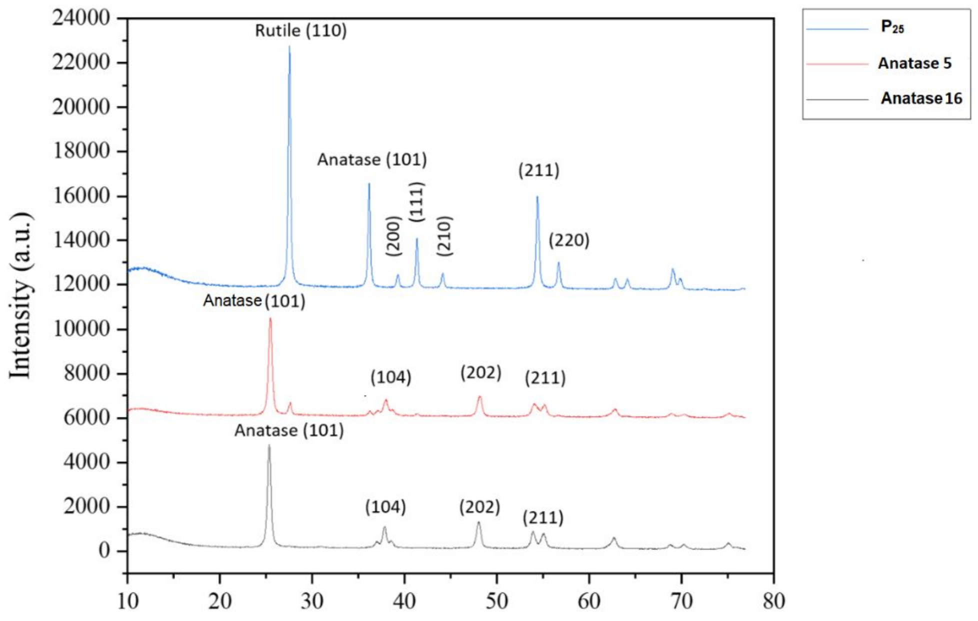

Figure S2. XRD analysis of (a) P25 (b) Anatase 5 (c) Anatase 16.

3. FT-IR Analysis of TiO_2 Plates

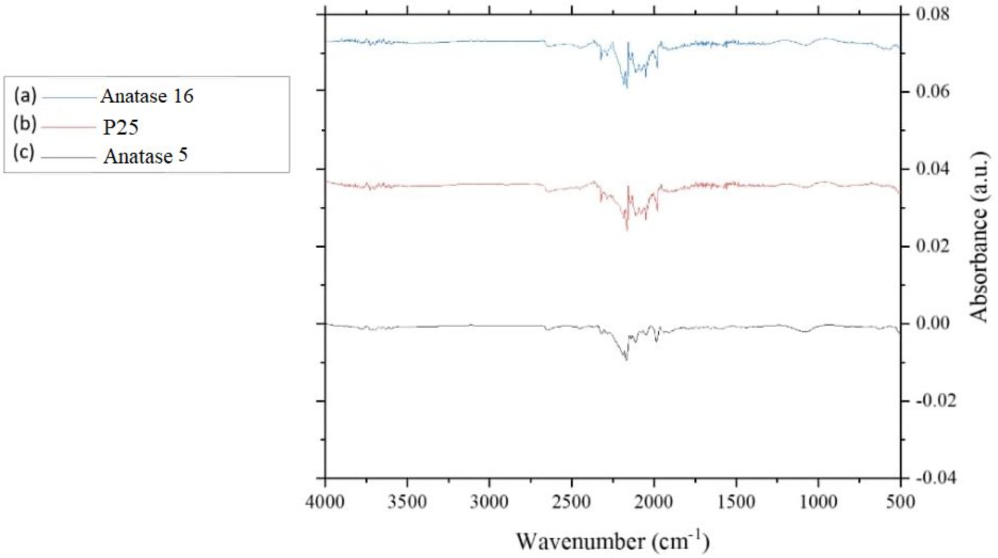

Figure S3. FT-IR analysis of (a) Anatase 5 (b) P25 (c) Anatase 16.

4. TiO_2 Particle Characterization

Table S1: TiO_2 Particle Characterisation

Photocatalyst	SBET [$m^2.g^{-1}$]	ξDW [mV]	ξGW [mV]	Eg [eV]
A5	115	-17.3	-15.1	3.38
A16	48	-17.2	-15.2	3.36
P25	50	-17.2	-15.1	3.4

5. Degradation information of target analytes

Table S2: Summary of percentage degradation and regression (R^2) of the target pharmaceuticals

**Pollutant type**	**Catalyst type**	**Percentage Degradation (%)**	**R^2**
NPX	Anatase 16	100	0.9866
	Anatase 5	100	0.9807
	P25	100	0.9923
SMX	Anatase 16	91	0.9924
	Anatase 5	76	0.9932
	P25	95	0.9952
MTF	Anatase 16	57	0.6869
	Anatase 5	65	0.7693
	P25	75	0.8703

6. Membrane bioreactor, construction start-up, operation, and physicochemical analysis of greywater

The lab-scale MBR was equipped with two ultrafiltration hollow polyvinylidene fluoride (PVDF) membrane modules (Pentair X-Flow, F5385; pore size = 0.03 µm; diameter = 8 mm; active filtration area = 0.044 m^2) connected in series (Figure S4). The reactor was inoculated with sludge from a full greywater treatment plant. The physicochemical parameters analyzed in raw greywater, effluent from the MBR, and sludge in the bioreactor included TSS, TDS, COD, Nitrogen (NH_3, NO_2, NO_3), pH, and conductivity. The Thermo Scientific Gallery™ Plus Discrete Analyzer (λ: 340-880 nm) was used to measure all parameters except solid measurements and COD. The COD_{Cr} (COD using $K_2Cr_2O_7$ as an oxidizing agent) was used for the raw greywater, while the COD_{Mn} (COD using $KMnO_4$ as an oxidizing agent) was used for effluent after MBR treatment due to the relatively lower COD content, usually less than 10 $mg.L^{-1}$. All measurements were carried out according to the American Public Health Association Standards Methods for the Examination of Water and Wastewater [52].The lab-scale MBR had a hydraulic retention time of 24 h and was aerated intermittently with a set dissolved oxygen (DO) concentration of ± 4 $mg.L^{-1}$. The reactor was run for three months to stabilize the system, and the influent was spiked with each of the target pharmaceuticals and run continuously at a concentration of 0.5 $mg.L^{-1}$.

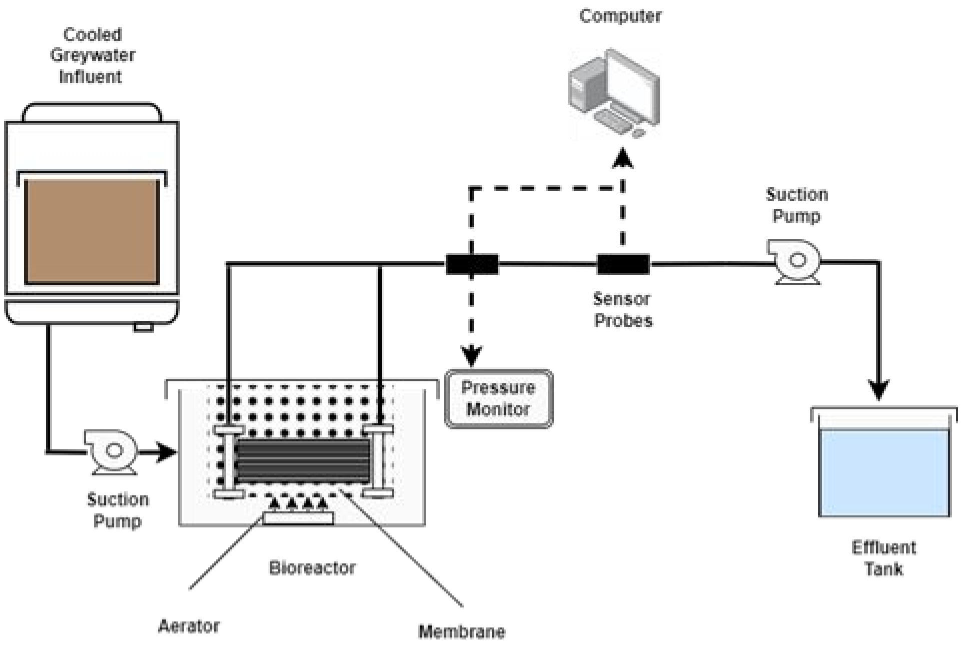

Figure S4. The setup of the lab-scale MBR used to produce model greywater treatment effluent.

Table S3: Physicochemical characteristics of greywater and MBR-treated greywater

**Physico-chemical parameter**	**Raw Greywater**	**MBR-treated Greywater**
Conductivity	0.00028- 0.38	0.002- 0.29
pH	7.0 – 8.3	7.2 - 8
TSS ($mg.L^{-1}$)	14.5 – 115.0	0
TDS ($mg.L^{-1}$)	227-327	240 - 402
sCOD ($mg.L^{-1}$)	12-73	1 - 25
tCOD ($mg.L^{-1}$)	51-670	1 - 36
NH_3 ($mg.L^{-1}$)	0.0085 -12.4	0.1 - 8.9
N-NO_2^- ($mg.L^{-1}$)	0.000041 – 1.16	0.002 - 0.8
N-NO_3^{2-} ($mg.L^{-1}$)	0.041 – 28.9	0.026 - 38.5

sCOD- Soluble COD, tCOD-Total COD.

7. Procedure for toxicology analysis

Two methods for determining toxicity were used as the procedures and results are highlighted as follows:

7.1 *Aliivibrio fischeri* assay

Aliivibrio fischeri assay is determined by the luminescence inhibition and was carried out according to the ISO Standard: ISO 11348-2: Water quality – Determination of the inhibitory effect of water samples on the light emission of *Vibrio fischeri* (Luminescent bacteria test) – Part 2: Method using liquid-dried bacteria. Liquid-dried bacteria (LCK 482, Hach Lange GmbH, Germany) were thawed and 0.5 ml of the reactivation solution was added. Afterward,

they were placed in a thermo-block at 15 °C. After reconstitution, bacteria were transferred with the use of a pipette into test tubes placed in a thermo-block set to 15 °C. Samples include raw greywater, MBR-treated greywater effluent, and greywater effluent treated with immobilised layers (Anatase 16, Anatase 5, and P25). The pH of the samples was not adjusted and no salts were added to the samples prior to toxicity assessment to avoid hyperosmotic effects. A solution of 20 $g.L^{-1}$ of sodium chloride (NaCl) was used as a control sample and zinc sulfate heptahydrate $ZnSH_{14}O_{11}$, was used as the reference sample. test, control, and reference samples were introduced into test tubes and then into a thermo-block to keep the test environment at a constant temperature of 15^0C. After the adaption of the bacteria, an initial luminescence test was carried out, and then the test, reference, and control samples were added to the bacteria at a 1:1 ratio of the bacterial suspension and sample. Luminescence tests were conducted at 15 and 30 min respectively. These tests were carried out in duplicates to ensure accuracy. the inhibitory effect of the samples was calculated according to ISO standard equations:

7.2 *Artemia salina* assay

Dried *Artemia* salina cysts (Easyfish, Czech Republic) were hatched in a cylindrical vessel filled with a 30 $g.L^{-1}$NaCl solution and subjected to a temperature of 24 °C and illuminated with an LED lamp for 26h. The hatched nauplii were then separated and placed into an aquarium. Samples were spiked with 30 $g.L^{-1}$ NaCl solution which was also used as a control and potassium dichromate ($K_2Cr_2O_7$) as a reference substance. Thereafter, 10 nauplii were transferred into a Petri dish using a micropipette and 10 ml of the test, reference, or control samples were added. Petri dishes were placed in an incubator (20 °C, 24h, LED lamp). Experiments were carried out in triplicates to confirm accuracy. After 24h, surviving nauplii were counted and the mortality rate was determined according to Abbott's formula as follows:

$$Percentage\ Death(\%) = \frac{Test - Control}{100 - Control} X\ 100 \qquad (4)$$

Table S4: Toxicological results of *Aliivibrio fischeri* assay

**CONTROL EXPERIMENTS**					
Control	Dilution level	fk15	fk15 mean	fk30	fk30 mean
1	2	0.93	0.894	0.903	0.876
2	2	0.872		0.879	
3	2	0.909		0.884	
4	2	0.864		0.838	
**TEST EXPERIMENTS**					
Sample	Dilution level	H15 (%)	H15 (%) mean	H30 (%)	H30 (%) mean
Raw greywater	2	92.4	92.6	96.7	97
	2	92.7		97.3	
MBR-treated greywater	2	30.2	29.4	36.1	36.2
	2	28.6		36.4	
Anatase 16	2	33.1	33	40.6	40.9
	2	32.9		41.2	
Anatase 5	2	33.7	35.4	41.7	41.1
	2	36		39.5	
P25	2	25.8	24.9	33.8	32.6
	**2**	**24**		**31.3**	

fkt - correction factor, which is a measure of intensity changes of control samples during the exposure time (15 or 30 min)

Ht – the inhibitory effect of a test sample after the contact time of 15 or 30 min, in percent.

8. Parameters for the detection of target analytes

The quantification of target analytes was performed by UPLC-MS/MS using a 1290 Infinity II LC system (Agilent Technologies) with a 6460 triple quadrupole mass spectrometer (Agilent Technologies) (conditions for QQQ MS in supplementary information – 5). A Zorbax Eclipse Plus C18 RRHD (2.1x50 mm; 1.8 µm) as a delay column and analytical column Zorbax Eclipse Plus C18 RRHD (2.1x100 mm; 1.8 µm) with a guard column Zorbax Eclipse Plus C18 pre-column (2.1 x 5 mm; 1.8 µm) at flow rate 0,40 mL/min were used for separation. Eluent A was aqueous 0.5 mM ammonium fluoride, and eluent B was CH_3OH. Gradient elution was used: 0 – 8 min 5 – 100 % B, held for 5.5 min 100 % B followed by a 13,50 - 14 min decrease to 5 % B and 3.5 min starting conditions before the next injection. The injection volume of samples was 100 µL. Quantification was achieved after LC separation with triple quadrupole using the positive ionisation mode. The ESI source with Agilent Jet Stream technology was operated under conditions given in (Table S5). The data recorded were processed with the software Mass Hunter B.08.00. For quantification and confirmation, two MRM transitions were monitored for each analyte in dynamic MRM mode for NPX 231,1→**185,1**,170,07; MET 130,1→**60,1**, 71,1 and SMX 254 → **92**, 156.

Source parameters for ESI source with Agilent Jet Stream Technology

Table S5: Source parameters for ESI source with Agilent Jet Stream Technology

**Parameter**	**value(+)**	**value (-)**
Gas temperature	230	230
Gas flow	8	8
Nebuliser	40	40
Sheath Gas Heater	380	380
Sheath Gas Flow	12	12
Capillary	3000	3000
VCharging	300	900

Conditions for QQQ Mass Spectrometer

Table S6: Conditions for QQQ Mass Spectrometer

Analyte	ISTD	Prec Ion MS1 Res	Prod Ion MS2 Res	Frag (V)	CE (V)	Cell Acc	Ret Time(min)	Ret Window	Polarity
NPX	No	231.1	185.1	116	12	4	7.59	2.0	Positive
NPX	No	231.1	170.1	116	28	4	7.59	2.0	Positive
SMX	No	$_{25}$4	156.0	113	12	4	5.31	1.5	Positive
SMX	No	$_{25}$4	92.0	113	24	4	5.31	1.5	Positive
MTF	No	130.1	71.1	98	24	4	1.05	3.0	Positive
MTF	No	130.1	60.1	98	12	4	1.05	3.0	Positive

9. Ionisation (ESI) and detection parameters of UHPLC–HRMS/MS

Table S7: Ionization (ESI) and detection parameters of UHPLC–HRMS/MS analysis for detection of transformation products of pharmaceuticals

**Mass spectrometer**	**Q-Exactive Plus™**
Ionisation	ESI +/–
Sheath/auxiliary gas (N_2)	45/10 arb. u.
Capillary temperature	320 °C
Heater temperature	300 °C
Spray voltage	3,5 kV
S-lens value	55
**Detection conditions: *Full MS* acquisition mode**	
Resolution	70 000 FWHM
Acquisition speed	3 Hz
Mass range *m/z*	80–1 200
**Detection conditions: *PRM* acquisition mode**	
Resolution	17 500 FWHM
Acquisition speed	12 Hz
Mass range *m/z*	50–*m/z* fragmented ion [+ 10 *m/z*]

10. Detected TPs identified during the degradation process

Table S8: Detected TPs for degradation of NPX, SMX and MTF

Compound	Elemental Formula	Exact Mass (*fullMS*)
**NPX**	$\mathbf{C_{14}H_{14}O_3}$	**231.1016 ($\mathbf{[M+H^+]}$)**
TP1	$C_{13}H_{14}O_3$	219.1016 ($[M+H^+]$)
TP2	$C_{13}H_{12}O$	185.0961 ($[M+H^+]$)
TP3	$C_{13}H_{14}O$	185.0972 ($[M-H^-]$)
TP4	$C_{13}H_{14}O_2$	201.0921 ($[M-H^-]$)
TP5	$C_{13}H_{12}O_2$	199.0765 ($[M-H^-]$)
TP6	$C_{11}H_{10}O$	157.0659 ($[M-H^-]$)
TP7	$C_{13}H_{12}O_3$	215.0714 ($[M-H^-]$)
**SMX**	$\mathbf{C_{10}H_{11}N_3O_3S}$	**254.0594 ($\mathbf{[M+H^+]}$)**
TP1	$C_{10}H_{11}O_4N_3S$	270.0543 ($[M+H^+]$)
TP2	$C_4H_6ON_2$	99.0553 ($[M+H^+]$)
TP3	C_6H_7N	92.0506 ($[M-H^-]$)
TP4	$C_6H_7NO_2S$	158.0270 ($[M+H^+]$)
TP5	$C_{10}H_{10}O_4N_2S$	255.0434 ($[M+H^+]$)
TP6	$C_{10}H_9N_3O_5S$	284.0336 ($[M+H^+]$)
TP7	$C_{20}H_{18}N_6O_6S_2$	503.0802 ($[M+H^+]$)
**MTF**	$\mathbf{C_4H_{11}N_5}$	**130.1087 ($\mathbf{[M+H^+]}$)**
TP1	$C_3H_9N_5$	116.0931 ($[M+H^+]$)
TP2	$C_3H_5N_5$	112.0618 ($[M+H^+]$)
TP3	$C_4H_7N_5$	126.0774 ($[M+H^+]$)
TP4	$C_2H_6N_4O$	103.0614 ($[M+H^+]$)

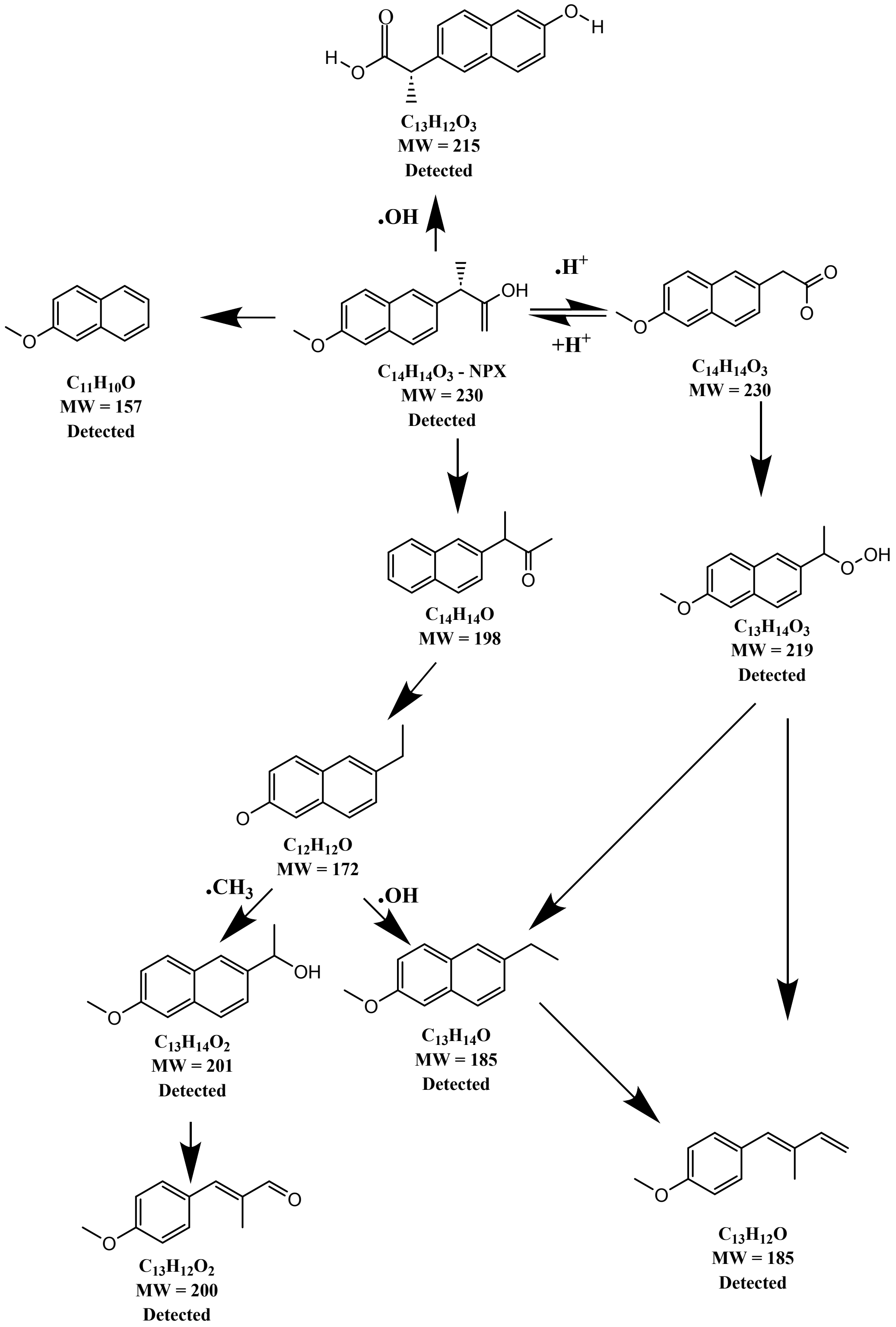

Figure S5: Proposed mechanism involved in the photocatalytic degradation of NPX.

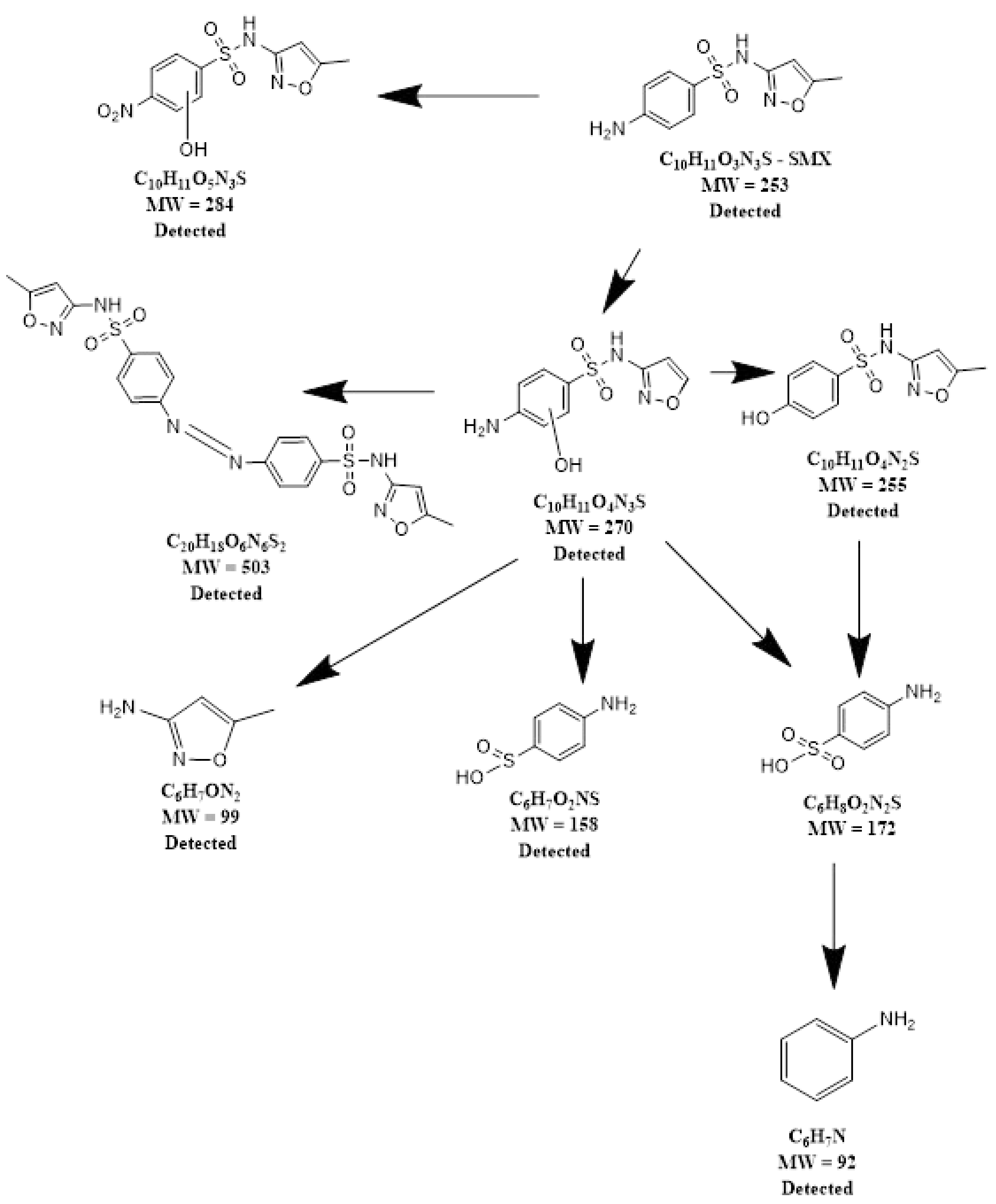

Figure S6: The proposed mechanism involved in the photocatalytic degradation of SMX.

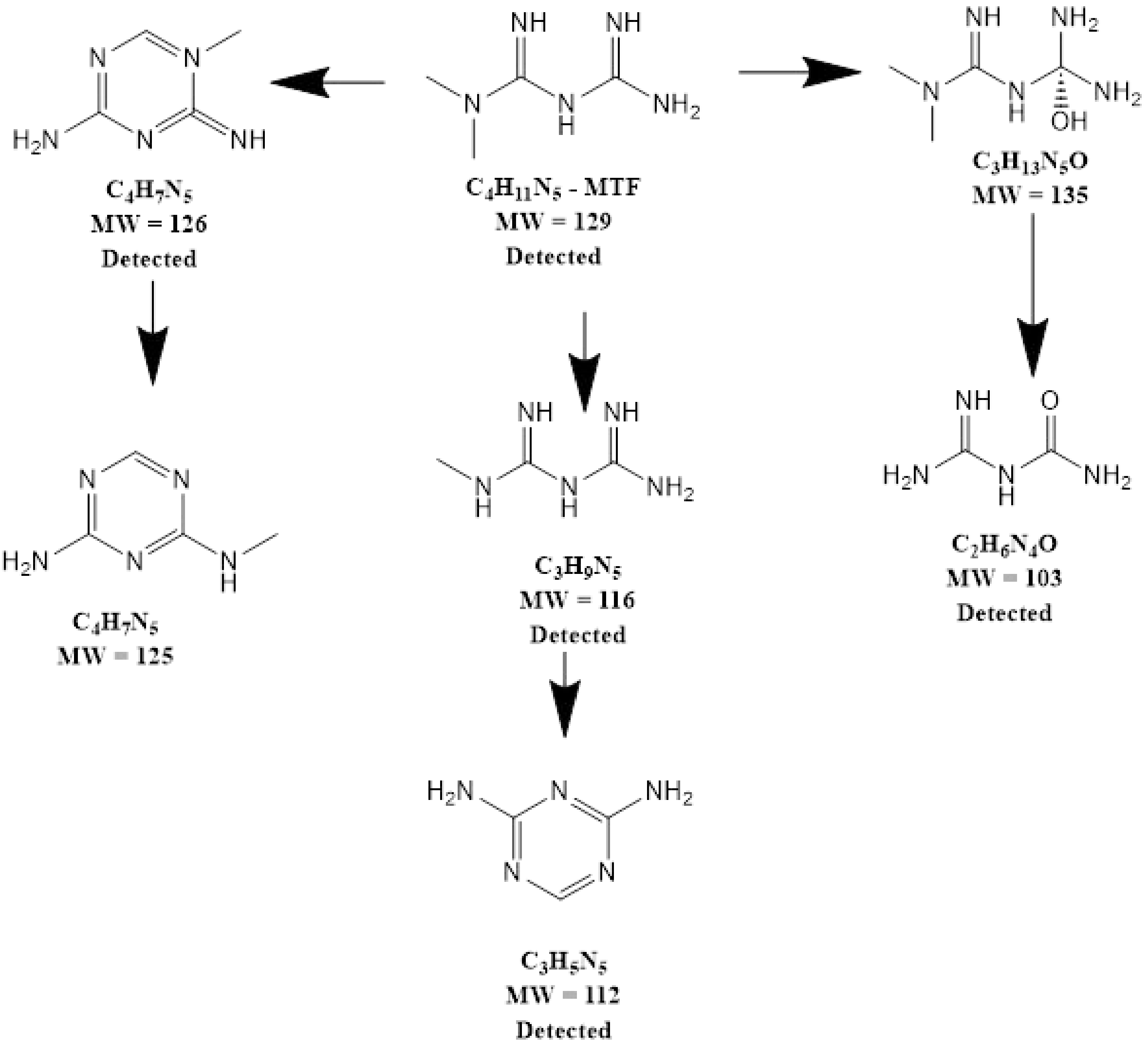

Figure S7: Proposed mechanism involved in the photocatalytic degradation of MTF.

11. Reusability of TiO_2 Layers in Photocatalytic Degradation

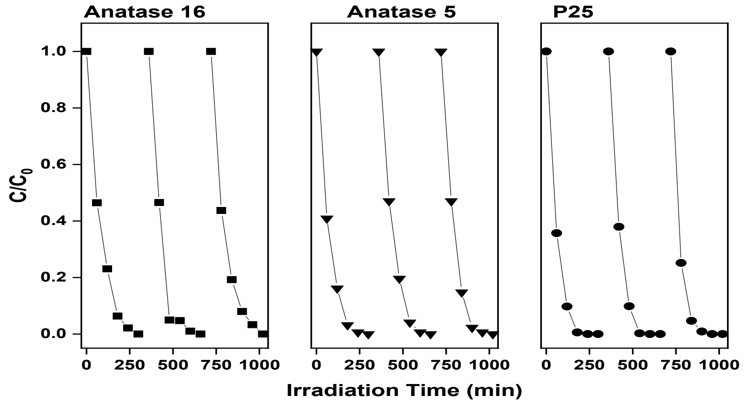

A

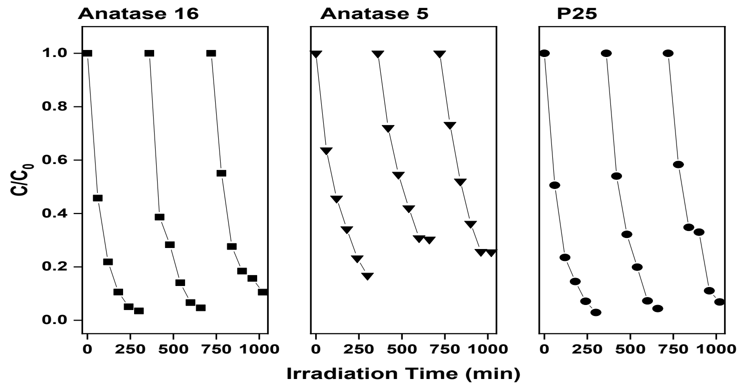

B

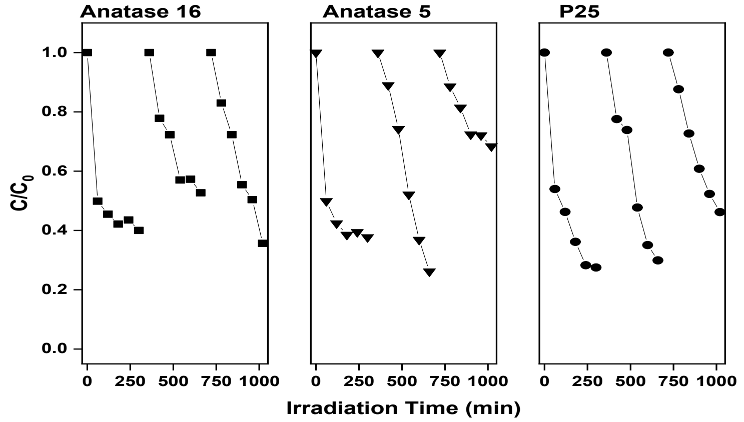

C

Figure S7: Photocatalytic degradation of NPX (A), SMX (B), and MTF (C) in three cycles each for Anatase 16, Anatase 5, and P25 TiO_2 layers. -rates of reaction
